# Stakeholder-informed pragmatic trial protocol of the TabCAT-BHA for the detection of cognitive impairment in primary care

**DOI:** 10.1186/s12875-024-02544-9

**Published:** 2024-08-06

**Authors:** Alissa Bernstein Sideman, Huong Q. Nguyen, Annette Langer-Gould, Eric A. Lee, Soo Borson, Ernest Shen, Elena Tsoy, Mayra Macias, Collette Goode, Katherine Rankin, Joel Kramer, Katherine L. Possin

**Affiliations:** 1 Department of Neurology, University of California, San Francisco, San Francisco, USA; 2 Global Brain Health Institute, University of California, San Francisco, San Francisco, USA; 3 Department of Research and Evaluation, Kaiser Permanente Southern California, Southern California Permanente Medical Group, 100 S. Los Robles Avenue, 2nd Floor, Pasadena, CA 91101 USA; 4 Department of Health Systems Science, Kaiser Permanente Bernard J Tyson School of Medicine, Pasadena, USA; 5 Department of Neurology, Los Angeles Medical Center, Southern California Permanente Medical Group, Los Angeles, USA; 6 Department of Clinical Sciences, Kaiser Permanente Bernard J Tyson School of Medicine, Pasadena, USA; 7 Department of Internal Medicine, Southern California Permanente Medical Group, West Los Angeles Medical Center, Los Angeles, USA; 8 Department of Family Medicine, University of Southern California Keck School of Medicine, Los Angeles, USA

**Keywords:** Cognitive impairment, Dementia, Detection, Primary care, Digital cognitive assessments

## Abstract

**Background:**

Cognitive impairment and dementia are frequently under-recognized. Health system strategies anchored in primary care are essential to address gaps in timely, comprehensive diagnosis. The goal of this paper is to describe the adaptation of a tablet-based brain health assessment (TabCAT-BHA) intervention and the study protocol to test its effectiveness in improving the detection of cognitive impairment, including dementia.

**Methods:**

This mixed-methods, pragmatic, cluster randomized, hybrid effectiveness-implementation trial is being conducted in two 18-month waves with 26 Kaiser Permanente Southern California primary care clinics, with 13 serving as intervention clinics and 13 as usual care clinics. Patients 65 years and older with memory concerns (*n* ~ 180,000) receiving care at the 26 clinics will be included in the analyses. Primary care clinics are provided the following practice supports as part of the TabCAT-BHA intervention: brief education and training on neurocognitive disorders and study workflows; digital tools to assess cognitive function and support clinician decision making and documentation; and registered nurse support during the work-up and post-diagnosis periods for primary care providers, patients, and families. The intervention was adapted based on engagement with multiple levels of clinical and operational leaders in the healthcare system. Effectiveness outcomes include rates of cognitive impairment diagnosis in primary care and rates of completed standardized cognitive assessments and specialist referrals with incident diagnoses. Implementation outcomes include acceptability-appropriateness-feasibility, adoption, and fidelity.

**Results:**

We identified seven themes organized by system-, provider-, and patient-level domains that were used to adapt the TabCAT-BHA intervention. Accordingly, changes were made to the provider education, diagnostic work-up, and post-diagnostic support. Results will be reported in fall of 2027.

**Conclusions:**

Our engagement with multiple primary and specialty care clinical and operational leaders to adapt the TabCAT-BHA intervention to these primary care clinics has informed the protocol to evaluate the intervention’s effectiveness for improving the detection of cognitive impairment, including dementia, in an integrated healthcare system.

**Trial Registation:**

Clinicaltrials.gov: NCT06090578 (registered 10/24/23).

**Supplementary Information:**

The online version contains supplementary material available at 10.1186/s12875-024-02544-9.

## Introduction

Early diagnosis of neurocognitive disorders is potentially highly beneficial for patients and families, yet cognitive impairment (CI) and dementia are frequently under-recognized. [1, 2] Health system strategies, especially efficient, scalable, and sustainable practice support models anchored in primary care, are essential to address gaps in timely, comprehensive diagnosis. [3–5] Studies of the Medicare annual wellness visit, which requires detection of CI if present, have found inconsistent effects on detection, [6, 7] and very few published studies address other ways to improve detection of CI in primary care. [8–12] The need is particularly acute among underserved populations for whom incomplete and delayed diagnosis are more prevalent. Our recent population-based study highlighted significant diagnostic disparities in that Black and Hispanic Medicare beneficiaries were less likely than White beneficiaries to be diagnosed in the early stages of cognitive decline, defined as a first diagnosis of mild cognitive impairment (MCI) versus dementia. [13]

The TabCAT Brain Health Assessment (TabCAT-BHA) intervention is an approach to efficiently and accurately support primary care providers (PCPs) to detect CI and complete important components of care. Following identification of a memory concern, the PCP requests back-office staff to complete the TabCAT-BHA, a 10-minute tablet-based assessment. The results are automatically available to the PCP in the electronic medical record (EMR) along with guidance on next steps to complete the work-up, diagnosis disclosure and post-diagnosis support. The TabCAT-BHA assessment is highly accurate for the detection of MCI and dementia due to both typical and atypical neurodegenerative syndromes among English and Spanish speakers. [14–16] Previous pilot work implementing an earlier version of this intervention found that streamlining workflow and providing EMR-integrated guidance were crucial for acceptability and adoption. [17]

**Fig. 1:**
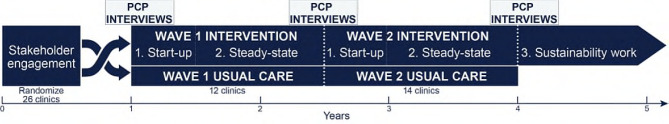
TabCAT-BHA Trial Design

The objective of this paper is to describe the stakeholder-driven adaptation of the TabCAT-BHA intervention and the protocol for a pragmatic cluster randomized, hybrid effectiveness-implementation trial of the intervention for the detection of CI, including dementia, in a large healthcare system, where we had previously identified care improvement opportunities. [18] The TabCAT-BHA trial is being conducted in two 18-month waves with 26 Kaiser Permanente Southern California (KPSC) primary care clinics that serve a highly diverse patient population in the greater Los Angeles area (Fig. 1). We hypothesize that use of the intervention will be associated with an increase in the rate of cognitive disorder diagnoses in the primary care intervention clinics and concurrently, increased use of standardized cognitive assessment with incident diagnoses and appropriate referrals to memory specialists in the total sample and in two priority subgroups, Black/African American and Hispanic/Latino patients.

## Methods

### Study setting

KPSC is a large integrated healthcare system providing care to over 4.7 million members who are broadly representative of the racially, ethnically, and socioeconomically diverse population of Southern California, including 31.9% of members living in the most socially vulnerable neighborhoods. [19] The 26 primary care clinics have a median of 18 providers and median panel size of 2054, serving over 180,000 patients 65 years and older (Blacks/African-Americans: 20%, Hispanics/Latinos: 35%, Asians/Pacific Islanders: 14%). Although a routine practice for many PCPs at these clinics has been to refer patients with memory concerns to geriatric memory clinic for work-up and diagnostic disclosure, this practice is not sustainable with the system’s large and growing aging population.

### Research ethics approval

The Institutional Review Boards of KPSC (#13451) and UCSF (23-40220) approved a waiver of written consent for the trial (clinicaltrials.gov: NCT06090578). Verbal consent was obtained from the physician and administrative leaders who participated in the site visit interviews.

### Randomization

We performed cluster-randomization within matched pairs of clinics by wave. The pairs were generated via non-bipartite matching to identify the set of unique pairs that minimized the sum of pairwise Mahalanobis distances across the matched clinics. [20, 21] Matching variables included number of providers, median panel size, percentage of members self-identified as Black/African American, Hispanic/Latino or Asian/Pacific Islander, baseline rate of CI diagnoses, rates of telehealth encounters, and percentage of members who were 65 + in the clinic. Thirteen clinics were randomized to receive the intervention and 13 to usual care.

### Eligibility criteria

Consistent with pragmatic trial principles, [22, 23] all patients seen at least once across the 26 clinics will be included and the primary analyses will use data extracted from the EMR. There are no exclusion criteria, except that patients who complete the TabCAT-BHA must be adults (18+), and we will limit our primary analyses to age 65+.

### Intervention description

**Fig. 2:**
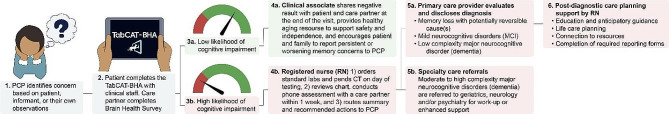
Adaptation of the TabCAT-BHA Intervention

The adapted TabCAT-BHA intervention (Fig. 2) described here reflects extensive stakeholder engagement efforts that are detailed in the subsequent sections. The core functions of this workflow are expected to be similar across clinics, but some logistical elements will be further adapted as needed.

In response to memory concerns reported by the patient or a family member or suspected by the PCP, the PCP requests that the TabCAT-BHA be completed by a bilingual clinical associate in the patient’s preferred language (Spanish or English) on the spot or scheduled for a future visit. Non-English and non-Spanish speakers are not eligible for the TabcAT-BHA intervention and are directly referred to the geriatric memory clinic. The TabCAT-BHA includes a 10-minute tablet-based assessment that evaluates memory, executive, and language skills, mood (PHQ-9), and a 3-minute informant survey which captures behavioral symptoms and changes from baseline. Patients classified as having a low likelihood of CI on the TabCAT-BHA are informed of the results at the end of the visit and are provided with print and web-based resources to support healthy aging, safety, and independence, and encouraged to notify their PCP if they have persistent or increasing cognitive concerns.

For patients classified as having a high likelihood of CI on the TabCAT-BHA, based on performance that is *≥* 1.5 standard deviations below a demographically-corrected expected mean (age, sex, education and testing language), [14] a registered nurse (RN) places an order for a battery of standard lab tests to be completed by the patient at the end of the visit and pends a head CT order for the PCP to approve in order to rule out potentially treatable causes of CI. The patient is also asked to nominate a family or friend who knows them well to provide information on how they are functioning at home. The RN then follows up with the family or friend via phone within a week to complete a functional assessment [24, 25] and triangulates the findings with the TabCAT-BHA results and chart review to arrive at a preliminary classification of mild or major neurocognitive disorder (MCI or dementia). The RN may also address any urgent needs identified during this first contact with the family.

If the patient’s preliminary classification is memory loss possibly due to potentially reversible causes, MCI or low complexity dementia, the RN routes the assessment summary to the PCP with guidance on next steps for diagnosis disclosure. For patients with moderate to high complexity dementia, the RN pends a specialty referral to either geriatrics, neurology, or psychiatry for the PCP to approve, depending on the complicating issue(s). Patients presenting with behavioral and psychological symptoms of dementia (e.g. hallucinations, delusions), motor disorders or other complicating neurological conditions, poorly controlled psychiatric illnesses, severe substance use, or patients who are kinless or have complicating family dynamics are considered to have moderate to high complexity dementia. After reviewing the results, the PCP is prompted to disclose a new CI diagnosis with the patient and family and has the option to do a warm hand-off back to the RN for a post-diagnosis support call. On this call, the RN engages in near and medium-term care planning, e.g. education, anticipatory guidance, and navigation to resources to support the patient and family. Should the patient or family needs exceed the capacity of the RN and the PCP, the patient is referred to social medicine, geriatrics, other specialty care or supportive services. The full duration of the intervention will vary but is expected to be approximately 3–4 months from concern to diagnosis disclosure for most patients.

Training for the RN role included completion of self-paced online training courses from Dementia Care Aware (Cognitive Health Assessment) and the UCSF Care Ecosystem, shadowing geriatric memory clinic team members, and participation in weekly interactive case conferences with dementia specialists.

**Implementation outcomes** follow the Proctor et al. [26] framework (Table 1 and Supplemental Table 1) and include: (1) Acceptability, Appropriateness, and Feasibility: Primary and specialty care stakeholders will be satisfied with the intervention and its fit to their practices based on their pre- and post-implementation feedback derived from qualitative interviews, and patients referred to TabCAT-BHA will find the assessment feasible based on high (> 80%) completion rates; (2) Adoption: percent of PCP in each clinic making at least one TabCAT-BHA referral; (3) Fidelity: percent of patients who complete the TabCAT-BHA for whom each of the six components of the intervention are also completed as described below. Primary fidelity analyses will be completed based on patients referred during the last 3 months of the 12-month steady state period to allow clinics sufficient time to adapt to the workflows.

**Table 1:** TabCAT-BHA trial implementation and effectiveness outcomes

Implementation Outcomes	Effectiveness Outcomes
**Acceptability**,** Appropriateness**,** Feasibility**: • Primary and specialty care leaders and front-line clinician satisfaction and fit of TabCAT-BHA intervention with their clinic practices through qualitative pre- and post-implementation interviews. • % of patients referred to TabCAT-BHA who completed the assessment. **Adoption**: • % of PCPs in each clinic who referred at least one patient to the TabCAT-BHA intervention and the mean and median use by provider, adjusted to the size of the 65 + patient panel. **Fidelity**: • % of patients who had six work-up components completed (*n* = 200 randomly selected records from the last 3 months of each wave). ✓ Documented concern ✓ Evaluated for reversible causes ✓ Identified and involved a care partner ✓ Documented and disclosed a cognitive diagnosis ✓ Provided appropriate educational or community resources to the patient ✓ Made appropriate referrals	**Primary**: Rate of patients with at least one cognitive impairment diagnosis documented in the medical record by any PCP during the 12-month steady state period at the clinic overall and stratified by race/ethnicity. (Goal: increase by 30%) **Secondary** • Rate of patients who have a documented standardized cognitive assessment performed in the primary care clinic within 4 months of an incident cognitive impairment diagnosis. (Goal: increase by 100%) • Rate of patients who have a referral to geriatrics, neurology, or psychiatry within 4 months of an incident diagnosis. (Goal: increase by 30%)

### Intervention fidelity

We will monitor and seek to maximize PCP adoption and fidelity to the intervention and will provide regular audit and feedback to the clinics and individual PCPs. Each month during the steady state implementation phase, the project manager will select 5 patients from each clinic who completed the TabCAT-BHA to review and document fidelity to the six intervention components: (1) documented cognitive concern, (2) evaluated for reversible causes, (3) identified and involved a care partner, (4) documented and disclosed a cognitive diagnosis, (5) provided appropriate educational or community resources to the patient, and (6) made appropriate referrals. Gaps in adoption and fidelity will be used to guide PCP engagement and education and further adaptations as needed.

### Effectiveness outcomes

The primary outcome is the Diagnosis Rate: the rate of patients with at least one CI diagnosis documented in the medical record by any PCP during the 12-month steady state period at the clinic overall and stratified by race/ethnicity (Blacks/African Americans and Hispanics/Latinos) (Table 1). Secondary outcomes are the Standardized Cognitive Assessment Rate: the rate of patients who have a documented standardized cognitive assessment performed in the primary care clinic within 4 months of an incident CI diagnosis, and the Referral Rate: the rate of patients referred to geriatrics, neurology, or psychiatry within 4 months of an incident diagnosis. Outcomes will be examined in the entire clinic population with primary analyses including only patients ages 65+.

### Power calculation

In a pilot of the TabCAT-BHA intervention in an academic family medicine clinic, we found a 40% increase in CI codes in the year after implementation. Due to the diversity of the primary care clinics involved in this trial, we projected a more conservative increase (30%) in rates of cognitive diagnoses for the total sample and for the two subgroups of Black/African American and Hispanic/Latino patients. At this effect size, and assuming intraclass correlation coefficients between 0.002 and 0.004, our power to detect a treatment effect at least that large will be in the range of 0.82–0.98 in the total sample, with similar ranges for the subgroups.

### Data collection methods

#### Pre and post implementation stakeholder engagement

We completed visits to the six intervention primary care clinics from February-March 2023 and will plan to do the same for the second wave of seven clinics in late 2024. The goal of these visits was to introduce the TabCAT-BHA, learn about current workflow around dementia detection and diagnosis and how the TabCAT-BHA might fit into clinic workflow, and to identify potential implementation facilitators and barriers. At least one physician and one administrative leader participated in interviews that lasted approximately 90 min each. HQN and MM co-led the interviews and ABS, a medical anthropologist, observed these visits and took fieldnotes. The interviews were digitally recorded and transcribed. These initial visits were complemented with multiple other stakeholder engagement touchpoints to provide clinician education and training, review and discuss processes and secure agreements on the workflows (Supplemental Table 2). The discussions from these meetings also informed workflow adaptations beyond the initial clinic visits. Follow-up interviews will be performed with the clinics towards the end of the implementation phase to glean insights on implementation barriers and facilitators as well as acceptability, appropriateness, feasibility and other factors associated with sustainability.

#### Effectiveness and implementation outcomes

The primary and secondary effectiveness outcomes will be extracted from the EMR using established cognitive impairment ICD-10 diagnostic codes, [17] documentation of any cognitive assessments from flowsheets and notes (keyword searches), and referrals to geriatrics, neurology, or psychiatry. The implementation outcomes of adoption will be extracted from the EMR and for fidelity, complemented with chart review.

### Analysis plan

#### Qualitative analyses

We used thematic analysis to analyze visit transcripts using ATLAS.ti. HQN and ABS developed an initial codebook based on fieldnotes from the visits. ABS then used the codebook to deductively code the transcripts and used inductive coding to identify additional codes in the data. HQN, ABS, and SB reviewed all codes during weekly team meetings and then these codes were subsequently developed into overarching themes. The team then met to discuss and revise code definitions and identify exemplary quotes from the data. This same approach will be repeated for the second wave of clinics and the follow-up (post-implementation interviews). We also relied on meeting notes and correspondence with the stakeholders in the ensuing months after the initial visits to refine the workflows.

#### Quantitative analyses

For Diagnosis Rate, we will compute difference-in-differences (DID) via generalized linear modeling. This will allow us to assess not only the between-group difference post-implementation, but also the changes pre- vs. post-implementation between the study arms, using a logit link function to estimate the odds of patients receiving a diagnosis vs. not, in the intervention versus usual care clinics. Treatment effects for the primary outcome will be evaluated in the entire sample, and separately in Black/African American and Hispanic/Latino patients using interaction terms. Analyses of the Standardized Cognitive Assessment and Referral Rates will use a similar general modeling framework, though may require log or linear link functions as appropriate. We will include all patients who are 65 years and older seen at least once in primary care across the 26 clinics. We will avoid selection bias by including patients only if they have at least 12 months of membership in KPSC before their assigned clinic’s implementation initiation. For patients who disenroll from KPSC, we will include their data up until the disenrollment date. Disenrollment, not due to death, is very low for this age cohort (3%). Adoption and fidelity will be analyzed using descriptive statistics.

## Results

This trial is being conducted between September 15, 2023 – September 14, 2026, and the primary findings will not be available until at least 2027. However, we describe below seven key themes derived from stakeholder engagement activities and organized by system-, provider-, and patient-level domains that were used to optimize the three foundational TabCAT-BHA intervention components (provider education and training; digital tools to assess cognitive function and support clinician decision making and documentation; and RN support during the work-up and post-diagnosis) for implementation at the KPSC clinics (Table 2).

**Table 2:** Insights from the pre-implementation engagement with primary care leaders: System-, provider-, and patient/family-level themes and corresponding adaptations to the workflows

Themes	Illustrative Quote(s)	Workflow Adaptations
**System-level**		
Time pressures	*“So*,* this is what really kind of scares me…It’s one thing to say*,* ‘Okay. You know*,* I’m worried about you. I’m going to do this work up*,*’ and whatever. But then*,* in that same visit – especially in that same visit*,* you’re already now falling behind. And now you’re going to say*,* ‘Oh*,* I have this tool. And I’m not actually able to diagnose you.’ That is a whole other visit to explain. I don’t have the time to do that*,* you know? Because that’s not just like*,* ‘Oh*,* you have a rash. Here’s some cream*,*’ or ‘Here’s an antibiotic.’ It’s not a quick examination. People are going to have a lot of questions. It requires a lot of discussion. So*,* in that respect*,* as much as I would love just point-of-care testing and evaluation and diagnosis*,* I don’t think it’s practical*,* not in the confines of our usual scheduling.* ” *(Site 1)*	• RNs order labs and imaging on behalf of the PCPs, provide a summary of the results to the PCP, and are available to provide post-diagnostic support to the patient and family. • PCPs schedule a dedicated face-to-face, video or phone follow-up appointment to disclose a new diagnosis with the patient and family even if a same-day TabCAT-BHA test is completed. • Limit the rate of false positives by classifying patients with a borderline score (-1z to -1.5z) as having a low likelihood of cognitive impairment. • Defer to the PCP to identify which patients will complete the TabCAT-BHA. Proactive identification of patients at high risk for undiagnosed cognitive impairment (e.g., using algorithms based on EMR data) will be deferred until capacity increases and stakeholders are ready.
Importance of streamlining workflows	*“Our face-to-face visits are premium*,* right? We have to use them wisely. We would love to see them back over and over again*,* but then other people can’t get in. So*,* I think we just try to be thoughtful that way. Like if they did see their physician already*,* they have this score*,* and now they need labs*,* then our staff is great. They call them up and they say*,* ‘These labs have been ordered. Either fast or don’t fast. Your phone appointment is this day. Please have them completed.’ Then most of the time it works – that they’ve done it. And by the time they have the appointment with you*,* you’re reviewing the results and telling them next steps.* ” (Site 6) *“Maybe if you just give us the abnormals*,* it would be great. If it was normal*,* don’t even. Is there a diagnosis that we can play with*,* like ‘screening for memory loss’? I mean*,* we could say…that the test was either negative or positive…if they could put it on the diagnosis list*,* on the problem list. Because sometimes it’s not really a memory loss issue; it’s a concentration issue…But at least it has come up*,* and we have done our due diligence in trying to see if they do have MCI or not. And then*,* therefore*,* they would say*,* you know*,* ‘We performed this test*,* and it was negative’ — or maybe in two years*,* it might be positive. But at least we started here*,* and we did it…So then this way*,* we’re doing our due diligence and saying*,* ‘You know what? You’re passing these tests. It’s not that; it’s probably depression*,* or*,* Hey*,* you know*,* it is evolving. We’ll go on to the next step’.” (Site 5)*	• Depression assessment is included in the TabCAT-BHA visit • Labs and head CT only ordered (by RN under the PCP) during the TabCAT-BHA visit for patients who have high likelihood of cognitive impairment and therefore, reducing unnecessary testing for patients with a low likelihood. • Patients who have a low likelihood of cognitive impairment are informed of the results and provided healthy aging resources at the end of the TabCAT-BHA visit. • Efforts to synchronize follow-up appointment for patients and families to discuss work up results with the PCP to minimize delays and improve efficiency.
**Provider-level**		
Variation across PCPs’ approach to dementia assessment and care	*“It’s probably provider-dependent. Some people kind of just automatically refer to geriatrics. I usually will select a Mini-Mental Exam or something just to kind of get some kind of an assessment. But usually*,* honestly*,* even when I do it*,* if the family has a concern*,* usually we’ll let them do an eval with geriatrics unless they do so fantastic on the Mini-Mental that I tell them there’s really probably not a concern for that at this time.”* (Site 4) *“I think it’s variable. Yes. I think based on their training*,* where they’ve trained*,* and how much exposure they’ve had. Because some are comfortable*,* and they even feel comfortable prescribing meds. Then there are others that are like*,* ‘I don’t want to be the one to make the call and I’m not going to start any medicine if I don’t need to. I need someone who knows.’” (Site 6)*	• All PCPs at the intervention clinics are participating in the trial and are encouraged to refer patients with memory concerns for the TabCAT-BHA visit. • The TabCAT-BHA results are shared with PCPs and are available to specialists to inform their work-up should be patient be referred. • Smartphrases were developed to help guide PCPs and improve standardization on ruling out reversible causes, patient/family education and anticipatory guidance, and treatments.
Discomfort disclosing a new dementia diagnosis	*“I don’t think any of us feel comfortable diagnosing anyone with dementia. I think we can say ‘cognitive impairment’. We can say ‘a memory disorder screening’. But dementia? You know*,* because once that patient gets that diagnosis of dementia*,* that’s something that will stick with them. ” (Site 1)* *“A lot of our physicians haven’t been the ones to initiate treatment before or provide a thorough diagnosis. And to ask them to do that now when they are like*,* ‘I wouldn’t want someone to do that to my own grandma. I would not do that to other people’s grandmas.’ And so*,* I think what they would want is that*,* if there is a positive diagnosis*,* there’s a referral to geriatrics” (Site 2).*	• RNs provide PCPs a preliminary diagnostic classification of the patients’ cognitive impairment based on chart review and informant assessments along with disclosure guidance and scripts, and are also available to provide post-diagnostic support to the patient and family. • Although we are promoting the standard pathways, PCPs have the option to refer patients to specialists for further work-up or diagnostic disclosure.
**Patient/family-level**		
Involvement of family	*“A discussion around memory tends not to be as patient-focused. It’s usually more focused on whatever family member is there bringing the concerns. As I’m sure you all know*,* most of the memory concerns aren’t obvious for the patient…So*,* it does change the focus of the appointment. It becomes more whoever came with them-focused as opposed to patient-focused.” (Site 3)* *“So usually the main caregiver will come in and say*,* ‘They’re becoming very forgetful. They’re very repetitive. They forgot that they ate. They’re asking for dinner number two*,*’ you know. And then we’re like*,* ‘Okay*,* let’s do the workup.’ We do the whole dementia work*,* lab*,* the CT of the head*,* and then send them to Geriatrics.” (Site 5)*	• Identification of family member or care partner at the outset of the TabCAT-BHA visit and strong encouragement for a family member or care partner to attend the scheduled visit with the patient. • Leveraging family insights in assessing patients’ daily functioning to inform the nurse’s preliminary classification of the patient’s cognitive impairment.
Importance of post-diagnostic care and wraparound support	*“Is there follow-up? Because I love Primary Care and preventative medicine*,* but this project in particular*,* to me*,* is really*,* really anxiety-provoking because this is not a straightforward conversation in any means. And it’s going to have a lot of follow-up. And you mentioned access….And then email. So*,* it’s not even appointments. It’s these people are going to be emailing us. The kids of these parents are going to be emailing us. So*,* we’re going to send them an email saying*,* ‘Oh*,* you have a low likelihood*,*’ you know*,* however it’s scripted and stuff like that. And people are still going to send*,* ‘Well*,* what else can I do?’ or*,* ‘What does this mean?’ or*,* ‘How is this going to affect my other condition?’” (Site 1)*	• There are two touch points by the RN. The first occurs during and/or immediately after completion of the TabCAT-BHA with a designated family member. The second phone or video call with the patient and family member occurs after the diagnostic disclosure by the PCP. This time-limited post-diagnostic care planning call includes education, anticipatory guidance, life care planning and connection to resources as appropriate. • Patients and families who require more extensive post-diagnostic support will be referred to social medicine or the geriatric memory clinic.
Appropriateness for diverse patients	*“A lot of my patients*,* you know*,* they’re*,* like*,* not even sixth-grade education*,* and they repeat a lot of things just because they’re unsure —some don’t read… it might take them a little while to read it. Other things are*,* they’re just very insecure when it comes to anything having to do with rote memorization*,* and it might*,* you know*,* trigger different results that need to be taken into account.” (Site 5)*	• After-visit summaries are either printed out or sent via postal mail to patients and families in addition to being available on the patient portal. • To minimize the risk of over-detecting cognitive impairment in historically underserved populations, we guide PCPs on using additional sources of information and their judgment to arrive at a diagnosis, use a conservative cut-point to detect cognitive impairment that is adjusted for education and testing language, and are conducting regular case reviews within the inter-disciplinary team and with the PCPs for selected cases.

### System-level

#### Theme 1. Prioritizing cognitive concerns is a challenge, given the time pressures in primary care

All clinic leaders expressed concerns about how to fit the TabCAT-BHA into busy practices given existing time pressures and need to address many other health issues. For example, some noted that memory concerns are often raised at the end of a visit or are not the primary reason why a patient schedules an appointment; others explained that it is often the family who raises the concern either via email or during an appointment for other medical issues. Concerns about time pressures led to discussions about how best to streamline the work-up, which is reflected in Theme 2.

#### Theme 2. Streamlining workflows to maximize efficiency, continuity, and communications across providers

Clinic leaders offered strategies to streamline the workflow including ways to minimize the number of PCP touchpoints, embedding a routine depression assessment, using Smartphrases to support key components of the workflow, providing group education for patients following testing, and having a “pending” diagnosis submitted by the RN to help PCPs know how to follow-up with the patient.

### Provider-level

#### Theme 3. Variation across PCPs’ existing approaches to dementia assessment and care

We found wide variation across clinics, and PCPs in the same clinic, on how they approach cognitive assessments, diagnostic disclosure, and post-diagnostic care.

#### Theme 4. Discomfort disclosing a new dementia diagnosis

Clinic leaders expressed concerns about being responsible for disclosing a new dementia diagnosis, noting a lack of experience and comfort in delivering a diagnosis or a preference for more general language or deferring disclosure to specialists.

### Patient-level

#### Theme 5. Questions around how to involve family in eliciting and evaluating cognitive concerns

Clinic leaders highlighted the importance of hearing about cognitive concerns from family members, and the need to figure out how best to involve them. Issues identified included the need to standardize and streamline identification and engagement of care partners in the process, as well as responding to their need for support and respite.

#### Theme 6. Importance of post-diagnostic care and wraparound support

Clinic leaders expressed concerns about what would happen after detection of CI, asking for support and best practices for what to do after testing, how to reach out to and engage with patients, when to refer to specialists, and how to get the required labs and imaging completed.

#### Theme 7. Concerns about the appropriateness of the assessment and follow-up for diverse patients

Clinic leaders articulated an interest in ensuring that the assessment was appropriate for detecting CI in all patients, including those who have low education or cannot complete the cognitive testing for reasons other than CI. They were also concerned about what follow-up would look like and offered strategies such as giving information to patients via both paper handouts and the EMR, sending patients letters ahead of time describing the test, and providing group education about what test results mean.

## Discussion

A scalable and sustainable health systems-based approach to earlier detection of cognitive impairment (CI) in primary care requires extensive engagement with physician and administrative leaders and front-line clinicians. [17, 27] It also requires careful coordination with intersecting specialties (geriatrics, neurology, and psychiatry), and leaders of other related initiatives to improve dementia diagnosis and care for alignment and consistent messaging. We described the pre-implementation stakeholder engagement [28] activities that were critical to adapting the three core elements of practice support across the clinics as part of the TabCAT-BHA intervention: brief provider education and training on neurocognitive disorders and study workflows; digital tools to assess cognitive function and support clinician decision making and documentation; and RN support during the work-up and post-diagnosis periods for primary care providers, patients, and families.

The overarching theme derived from iterative stakeholder meetings was that PCPs want to provide the best brain health care possible within the constraints of limited time and gaps in expertise. Standardizing a workflow that responds to patients’ and families’ cognitive concerns, implements a validated assessment of cognition and function, minimizes demands on overstretched PCPs, and ensures that only the most complex patients are referred to specialty care, can address the widespread need to increase detection and diagnosis of cognitive disorders in older people. Notably, primary care leaders agreed that with the EMR smartphrase guidance coupled with the RN support during the work-up and post-diagnosis periods, most PCPs would be amenable to sharing a new diagnosis of MCI or low complexity dementia with patients and families. For patients with moderate to high complexity dementia, both primary and specialty care leaders agreed that depending on the need, the RN could pend a referral on behalf of the PCP for patients to receive further work-up, diagnosis disclosure and/or support from geriatrics, neurology and/or psychiatry, to streamline care for patients. Securing these agreements across the service lines was foundational for the intervention protocol and required many one-on-one and group conversations with the respective leaders, beyond the initial site visit.

Important concerns expressed by clinic leaders was perceived lack of readiness to absorb a rapid increase in diagnosis rates and potential risks of over-detecting CI, particularly among historically disadvantaged populations who might perform poorly on standardized testing due to reasons other than acquired CI, e.g. very low education. To mitigate these risks, the intervention encourages clinicians to use their judgment, and supports them by incorporating standardized informant reports and results from an RN consultation that evaluates cognitive, behavioral, and functional changes, as well as other clinical data regarding potentially reversible causes of CI. Our regression-based normative corrections adjust for patient education, testing language, age, and sex. We selected a conservative threshold for adjudicating high likelihood of impairment (1.5 standard deviations below an individual’s reference group mean); this contrasts with prior work where patients with intermediate scores of -1.5 to -1.0 also underwent further evaluation. [17] Monthly fidelity reviews will also help identify any care gaps that may be leading to over (or under) detection.

There are limitations of the TabCAT-BHA intervention for improving the detection of CI. As a result of our efforts to minimize false positive detection, patients may be falsely reassured of brain health when they have early-stage neurodegenerative disease. This limitation may become particularly urgent to address as we enter a new era of disease modifying therapies for Alzheimer’s disease that are most efficacious in the earlier stages. [29, 30] The intervention relies on PCPs to be alert to and formally identify patient- and family-raised memory concerns as the initiating step in the protocol. We explored other opportunities to identify patients at high risk for having undetected CI using validated algorithms, [31, 32] and sending pre-visit queries about their memory. However, since the health system’s resources are not yet aligned with the expected high volume of patients requiring evaluations, our protocol uses a conservative approach to initiating cognitive assessments. If the trial results are successful both in effectiveness at increasing diagnosis and acceptability to PCPs, we expect future opportunities for earlier and more inclusive detection, diagnosis, and brain health care.

### Electronic supplementary material

Below is the link to the electronic supplementary material.

Supplementary Material 1

## Data Availability

The data are available for use through collaboration with the KPSC study investigators under the conditions of sufficient funding by the requestor and data use agreements between all institutions that govern data access, storage, and short- and long-term use. Qualified researchers trained in human subject confidentiality protocols interested in collaborating with the KPSC study team can contact Dr. Huong Nguyen (huong.q2.nguyen@kp.org).

## Abbreviations

CI: Cognitive impairment
EMR: Electronic medical records
KPSC: Kaiser Permanente Southern California
PCP: Primary care provider
RN: Registered nurse
Tab-CAT BHA: Tablet-based brain health assessment
