# Supplementary material for: Stakeholder-informed pragmatic trial protocol of the TabCAT-BHA for the detection of cognitive impairment in primary care
Source: BMC Prim Care. 2024 Aug 6;25:286. doi: 10.1186/s12875-024-02544-9 (PMC11302096; doi:10.1186/s12875-024-02544-9)
Supplement: Supplementary file 1 — Supplementary Material 1 [file 12875_2024_2544_MOESM1_ESM.pdf]

Supplemental Table 1. TabCAT-BHA Trial Implementation Outcome Details

**Implementation outcome**	**Level of analysis**	**Theoretical basis**	**Other terms in the literature**	**Salience by implementation stage**	**Detect CID Measurement**
Acceptability	Individual provider<br>Individual patient	Rogers: ‘‘complexity’’ and to a certain extent ‘‘relative advantage’’	Satisfaction with various aspects of the innovation (e.g. content, complexity, comfort, delivery, and credibility)	Early for adoption<br>Ongoing for penetration<br>Late for sustainability	Y1-Y4: Qualitative pre- and post-implementation interviews with primary and specialty care leaders and front-line clinician to assess satisfaction and fit of TabCAT-BHA intervention with their clinic practices.<br><br>Y2-Y4: % of patients referred to TabCAT-BHA who completed the assessment.
Appropriateness	Individual provider<br>Individual patient<br>Organization or setting	Rogers: ‘‘compatibility’’	Perceived fit; relevance; compatibility; suitability; usefulness; practicability	Early (prior to adoption)	
Feasibility	Individual providers<br>Organization or setting	Rogers: ‘‘compatibility’’ and ‘‘trialability’’	Actual fit or utility; suitability for everyday use; practicability	Early (during adoption)	
Adoption	Individual provider<br>Organization or setting	RE-AIM: ‘‘adoption’’<br>Rogers: ‘‘trialability’’ (particularly for early adopters)	Uptake; utilization; initial implementation; intention to try	Early to mid	Y2-Y4: % of PCPs in each clinic who referred at least one patient to the TabCAT-BHA intervention and the mean and median use by provider, adjusted to the size of the 65+ patient panel.
Fidelity	Individual provider	RE-AIM: part of ‘‘implementation’’	Delivered as intended; adherence; integrity; quality of program delivery	Early to mid	Y3-Y4: % of patients who had six work-up components completed (n~200 records from the last 3 mos of each wave).
Implementation Cost	Provider or providing institution	TCU Program Change Model: ‘‘costs’’ and ‘‘resources’’	Marginal cost; cost-effectiveness; cost-benefit	Early for adoption and feasibility<br>Mid for penetration<br>Late for sustainability	Y4: Micro-costing and time and motion observations with clinical associates and nurses *(not described in the manuscript)*
Penetration	Organization or setting	RE-AIM: necessary for ‘‘reach’’	Level of institutionalization; spread; service access	Mid to late	Y5: Post trial completion
Sustainability	Administrators<br>Organization or setting	RE-AIM: ‘‘maintenance’’<br>Rogers: ‘‘confirmation’’	Maintenance; continuation; durability; incorporation; integration; institutionalization; sustained use; routinization;	Late	Y5: Post trial completion

Reference #22: Proctor et al. *Adm Policy Ment Health*. Mar 2011;38(2):65-76. TabCAT-BHA (Tablet-based Cognitive Assessment Test-Brain Health Assessment)

Supplemental Table 2. TabCAT-BHA Trial Pre-Implementation Adult Primary Care Stakeholder Engagement Activities

Engagement Activities	Goals	Key Insights
Kick-off virtual meeting with medical center internal and family medicine chiefs (Jan 2023; 60 mins)	• Reviewed project goals<br>• Discussed facilitators and potential barriers for implementation<br>• Sought advice on how to structure education for busy PCP practice<br>• Make email introductions to intervention site physician and administrative leaders	• Recognition of primary care's role and contributions to detecting and diagnosing cognitive impairment but realistic concerns<br>• Cautious optimism for tools and practice support for overwhelmed providers<br>• Avoid web-based training since providers will likely not participate. Chunk education into 15 min segments during standing clinic meetings
Introductory virtual meeting with physician and administrative leaders at each of the six intervention clinics (Feb-Mar 2023; 30 mins)	• Reviewed project goals, emphasizing close partnership on planned activities<br>• Address general implementation questions and concerns<br>• Scheduled on-site visit	• Reactions ranged from cautious optimism for tools and practice support for overwhelmed providers to enthusiastic embrace of the project since the health system had not invested attention in dementia care previously
On-site workflow discussion with clinic physician and administrative leaders at the six intervention clinics (Feb-Apr 2023; 90 mins) **Note: Quotes used to illustrate key themes were drawn from these recorded site visits**	• Reviewed current workflows in response to patient or family report of memory concern<br>• Demonstration of TabCAT-BHA<br>• Discussed how TabCAT-BHA could be integrated into current workflows	• Convergence on need for streamlined approach to scheduling TabCAT-BHA visit, informing patients of negative results, and development of standard scripting for all patient touchpoints.<br>• Concerns regarding variation in PCP preference and comfort level with disclosing a new dementia diagnosis
Brief education to adult primary care providers during standing meetings at the intervention clinics (Jul-Sep 2023; 15-30mins)	• Guidance on distinguishing mild vs. major neurocognitive disorders<br>• Discussed the anticipated new Alzheimer's treatments<br>• Brief introduction to TabCAT-BHA project	• Urgent health system focus on improving patient access to care and preparations for work stoppage during Q2/Q3 led to delays.<br>• Education provided by respected a primary care leader with geriatrics expertise and a translational science neurologist was well received and synchronized with FDA approval of Lecanemab
Staggered soft launches across six intervention clinics (Sep-Dec 2023)	• Adapt processes to local site needs, preferences, and constraints while retaining core functions.<br>• Actively engaged "early PCP adopters" to refine processes, ensuring PCP needs are met to enhance buy-in	• Secured agreements across primary care, geriatrics, neurology, and psychiatry on the diagnostic and disclosure pathways.<br>• Need to balance brevity and comprehensiveness in developing smartphrases to support PCP with disclosing a new cognitive impairment diagnosis
Clinician and staff training on workflows at the six intervention clinics (Jan-Mar 2024; 30-60 mins)	• Socialize and reinforce workflows with providers, back-office staff and other supporting units<br>• Adjust workflows and processes as needed	• Positive feedback regarding overall workflow from early PCP adopters though some reservations from several PCPs on whether a new diagnosis of low complexity dementia should sit with primary care.<br>• Refined processes to minimize delays in completing nurse assessments and patient follow-up visits with providers.

Abbreviations: TabCAT-BHA (Tablet-based Cognitive Assessment Test-Brain Health Assessment), PCP (primary care provider)
